# Proximity effects across oxide-interfaces of superconductor-insulator-ferromagnet hybrid heterostructure

**DOI:** 10.1038/s41598-018-22036-y

**Published:** 2018-02-27

**Authors:** C. L. Prajapat, Surendra Singh, D. Bhattacharya, G. Ravikumar, S. Basu, S. Mattauch, Jian-Guo Zheng, T. Aoki, Amitesh Paul

**Affiliations:** 1 Technical Physics Division, Bhabha Atomic Research Centre, Mumbai, 400085 India; 2 Solid State Physics Division, Bhabha Atomic Research Centre, Mumbai, 400085 India; 3 Homi Bhabha National Institute, Anushaktinagar, Mumbai, 400085 India; 4 Jülich Centre for Neutron Science (JCNS) at Heinz Maier-Leibnitz Zentrum (MLZ), Forschungszentrum Jülich GmbH, Lichtenbergstraße 1, D-85747 Garching b. München, Germany; 5 Irvine Materials Research Institute, University of California, Irvine, CA 92697-2800 USA; 6 Technische Universität München, Physik Department E21, Lehrstuhl für Neutronenstreuung, James-Franck-Straße 1, D-85748 Garching, Germany

## Abstract

A case study of electron tunneling or charge-transfer-driven orbital ordering in superconductor (SC)-ferromagnet (FM) interfaces has been conducted in heteroepitaxial YBa_2_Cu_3_O_7_(YBCO)/La_0.67_Sr_0.33_MnO_3_(LSMO) multilayers interleaved with and without an insulating SrTiO_3_(STO) layer between YBCO and LSMO. X-ray magnetic circular dichroism experiments revealed anti-parallel alignment of Mn magnetic moments and induced Cu magnetic moments in a YBCO/LSMO multilayer. As compared to an isolated LSMO layer, the YBCO/LSMO multilayer displayed a (50%) weaker Mn magnetic signal, which is related to the usual *proximity* effect. It was a surprise that a similar proximity effect was also observed in a YBCO/STO/LSMO multilayer, however, the Mn signal was reduced by 20%. This reduced magnetic moment of Mn was further verified by depth sensitive polarized neutron reflectivity. Electron energy loss spectroscopy experiment showed the evidence of Ti magnetic polarization at the interfaces of the YBCO/STO/LSMO multilayer. This crossover magnetization is due to a transfer of interface electrons that migrate from Ti^(4+)−*δ*^ to Mn at the STO/LSMO interface and to Cu^2+^ at the STO/YBCO interface, with hybridization *via* O 2*p* orbitals. So charge-transfer driven orbital ordering is the mechanism responsible for the observed proximity effect and Mn-Cu anti-parallel coupling in YBCO/STO/LSMO. This work provides an effective pathway in understanding the aspect of long range proximity effect and consequent orbital degeneracy parameter in magnetic coupling.

## Introduction

Multilayered structures with artificial oxide heterointerfaces have recently dominated the field of new states of matter, leading to novel functionalities^1,2^. Fundamentally, their unexpected properties are a consequence of the symmetry breaking and/or electronic reconstruction at the interface due to dissimilar oxide materials. Among the heterointerfaces showing interplay of different degrees of freedom, such as charge, spin, orbital and lattice, one may recall the case of the charge-transfer-driven orbital ordering and ferromagnetism in superconductor (SC)-ferromagnet (FM) interfaces of YBa_2_Cu_3_O_7_ (YBCO)/La_0.67_Ca_0.33_MnO_3_ (LCMO)^3^.

Apart from the aspects of usual high ferromagnetic transition temperature *T*_C_ and high spin polarization in the SC-FM system, superconductivity induced *suppression* of ferromagnetic ordering of LCMO and *proximity* induced spin-triplet superconducting state with induced Cu magnetic moments have added new dimensions to this field^4^. On the one hand, the suppression of Mn magnetic moments at the interface was studied by polarized neutron reflectivity (PNR)^5^. On the other hand, X-ray linear dichroism (XLD), X-ray absorption spectroscopy (XAS) and X-ray magnetic circular dichroism (XMCD) studies indicated that an orbital reconstruction of the Cu atoms associated with a charge transfer across the interface and an anti-parallel coupling between the YBCO-LCMO interface are basically responsible for the proximity effect^6–9^. All these phenomena had been explained in terms of the covalent bonding of Cu and Mn atoms *via* the oxygen atom, which also rely on the specific interface termination^3^. However, subsequent theoretical and experimental studies on YBCO/LCMO multilayers (MLs) have raised controversy between the orbital construction concept and the cases of induced Cu magnetic moments^10,11^. While the induced Cu magnetic moments have been shown to closely follow the temperature dependence of Mn magnetism, lack of any orbital reconstruction in such a system contradicts the proposed covalent bonding theory.

The key factor that is established behind the commonly observed proximity effect can be divided into two parts, above and below the superconducting ordering temperature (*T*_SC_), which bespeak that they may not be directly related. One part being the FM proximity effect, which develops at a temperature much higher than *T*_SC_ and is predicted to be a suppression of magnetism on the FM side with the formation a magnetic dead layer, the orbital reconstruction and charge transfer. Below *T*_SC_, aided by the conduction band of the FM, the other part is the SC proximity effect which can be described in terms of leakage of the spin-triplet Cooper pairs into the FM leading to lowering of the transition temperature in the SC. This obviously means that in case the FM-SC layers are interleaved with a band insulator, the leakage of Cooper pairs should stop and there will be an abrupt break in the long range order of the triplet spin-paring. The best way to test the validity of the covalent bonding model is therefore to interleave the YBCO and LCMO layers with a layer of insulating (I) SrTiO_3_(STO). In that case, we should neither expect any induced magnetism of the Cu atoms on the YBCO side nor any reduction of the Mn magnetic moments on the LCMO side. However, our recent findings on similar systems involving trilayers of FM(LCMO)-I(STO)-SC(YBCO) contradict the present understanding^12^. Based upon the PNR data we demonstrated earlier that there was lower magnetization on the ferromagnet side designated by the so called “dead layer”. Additionally, this dead layer was strongly affected by the thickness of the interleaved STO layer. The PNR experiment confirmed that there was either a tunneling of the Cooper pairs from the SC to the FM layer or a polarized charge transfer from the FM to the SC layer across STO. The possibility of such a reduction in magnetization could be charge-transfer driven orbital ordering *via* the Ti ions which often show signatures of extra charge. Ti^4+^ ions have no occupied 3*d* electrons in the ground states 3*d*^0^, and so there are no *d* electrons available for the induced spin polarization. However, for Ti^3+^ with 3*d*^1^, the situation can be quite different. Ti^(4+)−*δ*^ state with Ti partitioning between the two valency states has often been reported in other systems^13^.

In this paper, we present XMCD results from two MLs measured as a function of temperature, traversing through their *T*_SC_. One ML has no intervening STO layer between YBCO and La_0.67_Sr_0.33_MnO_3_ (LSMO) and the other ML has an intervening layer of STO in between. Firstly, our results confirm the usual existence of anti-parallel coupling between Mn magnetic moments and induced Cu magnetic moments in the YBCO-LSMO ML below *T*_SC_. Secondly, we did observe the expected decrease in the Mn XMCD signal around *T*_SC_ as we follow the strength of the L_3_ edge peak with temperature. Interestingly, even for the YBCO-STO-LSMO ML, a similar decrease in the Mn signal was observed although the Mn signal was weaker than that in the YBCO-LSMO ML. Thirdly, the reduction in the magnetic moment value was also supported by depth sensitive PNR data. Furthermore, the induced Cu magnetism, which was unidentifiable at 300 K, is shown to be anti-parallel to the Mn magnetism across *T*_SC_, at 100 K and 10 K. These two magnetic signals, one from Mn and the other from Cu, indicate either a case of tunneling of Cooper pairs or polarized charge transfer *via* Ti^(4+)−*δ*^ states across the band insulator. Lastly, electron energy loss spectroscopy (EELS) measurements at the Ti edge confirm the presence of Ti^(4+)−*δ*^ states within the STO layers. Thus a hybridization across the interfaces *via* O 2*p* orbitals is the possible physical process for the reduced effect of long range proximity.

## Sample Preparation

Three ML samples were prepared on TiO_2_-terminated STO (001) substrates by pulsed laser deposition method (PLD). The samples were labelled as S1, S2 and S3 with details shown below:

- S1: [YBCO(30 nm)/LSMO(30 nm)] _× *N* = 5_
- S2: [YBCO(30 nm)/STO(5 nm)/LSMO(30 nm)] _× *N* = 5_
- S3: [YBCO(30 nm)/STO(5 nm)/LSMO(30 nm)] _× *N* = 1_

Here, N is the number of repetitions of the bilayer or trilayer combinations. The growth process of these samples was similar to that reported elsewhere^12^. These samples were composed of stacked (YBCO)_*n*_ and (LSMO)_*m*_ layer units. The YBCO layer has a thickness of 26 u.c. (n = 26), the LSMO layer has 77 u.c. (m = 77 u.c.) and the nominal STO layer thickness has around 12 u.c.

## Results

### X-ray diffraction and magnetization measurements

**Figure 1:**
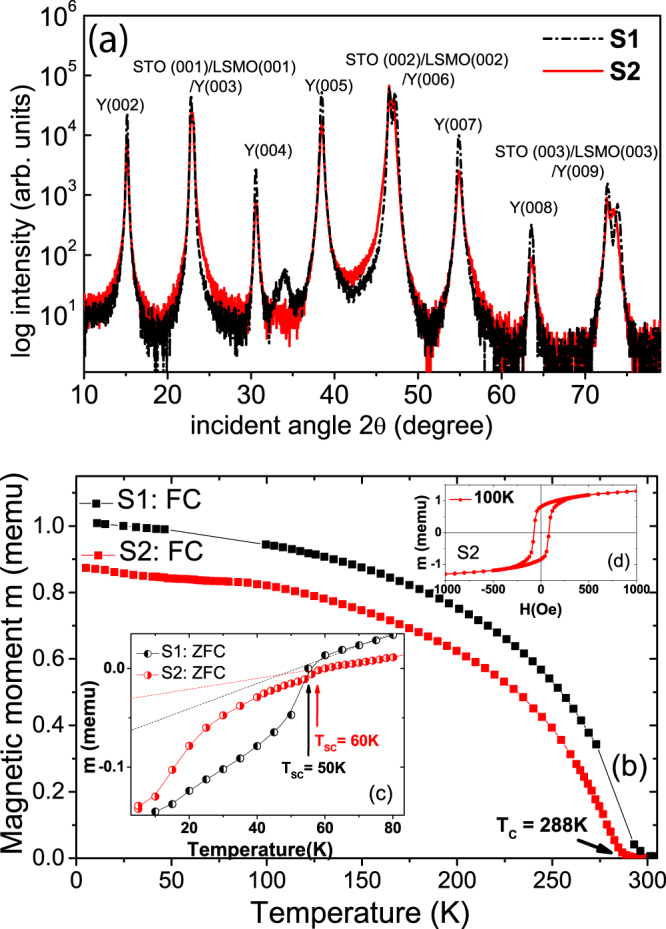
XRD and SQUID measurements of specimens S1 and S2. (**a**) XRD data from S1 and S2 grown on (001) STO substrates. (**b**) Field cooled curves as a function of temperature for the S1 and S2 MLs showing their ferromagnetic transitions. (**c**) The bottom inset shows the zero field cooled curves for both MLs. The typical kink in the curves indicate the superconducting transition temperatures *T*_SC_ = 50 K and *T*_SC_ = 60 K for S1 and S2, respectively. (**d**) The inset on the top right hand corner shows the hysteresis loop measurement at 100 K for the S2 ML.

Figure 1(a) presents the X-ray diffraction (XRD) spectra from the S1 and S2 MLs on STO substrates. The spectra show only 00l-type of diffraction peaks from the layers, suggesting a preferential (001) orientation growth of the MLs, *i.e*., the *c* axes of the MLs are perpendicular to the sample surface. Figure 1(b,c) show the macroscopic magnetization of S1 and S2 as a function of temperature and magnetic field under field-cooled (FC) (cooling field H_*FC*_ = 300 Oe) and zero-field-cooled (ZFC) conditions, respectively. A typical hysteresis loop at 100 K is also shown (top inset) for the sample S2 (Fig. 1(d)). S1 and S2 have similar magnetization behavior (Fig. 1(b)), i.e., FC magnetization decreases as the temperature increases. The FC data indicates that the LSMO layer in S2 has a Curie temperature T_*C*_ ≈ 288 K which is slightly lower than that in S1. The ZFC data (Fig. 1(c)) shows *T*_SC_ ≈ 50 K and *T*_SC_ ≈ 60 K for S1 and S2, respectively. Both of them are lower than the usual value of *T*_SC_ of YBCO (≈90 K), suggesting that the YBCO was under-doped.

### Transmission electron microscopy (TEM) measurements

#### Microstructure and composition

**Figure 2:**
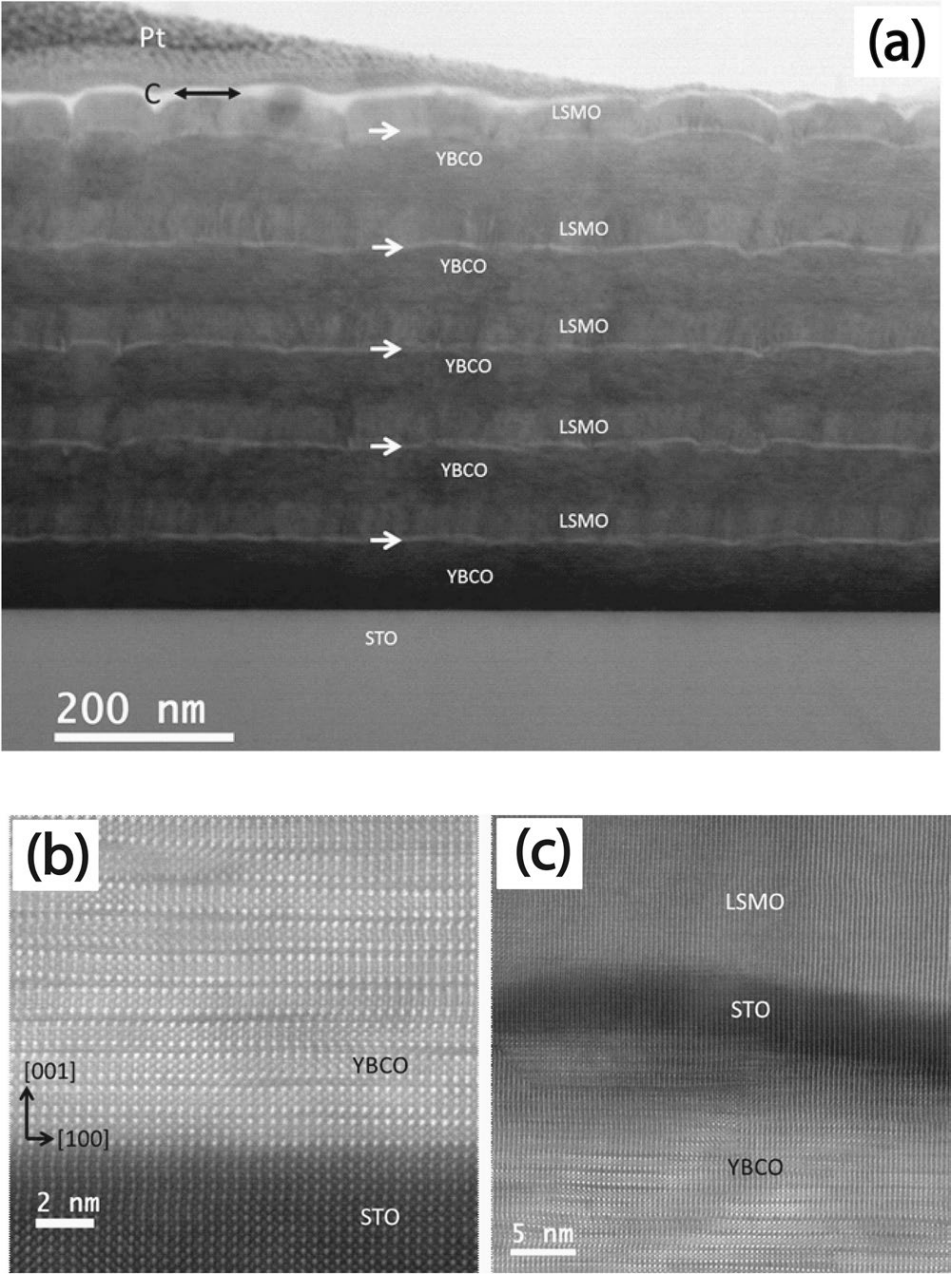
STEM measurements of specimen S2. (**a**) Bright-field STEM cross-sectional image of the specimen S2, showing trilayer repetitions of the stack on the STO substrate. (**b**) HAADF STEM (Z-contrast) image of the YBCO layer on the STO substrate. (**c**) LSMO, STO and YBCO trilayer where a thin STO layer (about 5 nm) is sandwiched between LSMO and YBCO.

Figure 2(a) shows the morphology of the LSMO/STO/YBCO multilayers grown on a STO substrate. This bright field scanning transmission electron microscopy (STEM) image clearly shows the contrast difference between the labeled LSMO/STO/YBCO layers, where the STO layers indicated by white arrows are thin (about 5 nm). Thin carbon layer and Pt protection layers are visible as well.

The microstructures of the specimen were also investigated by high resolution transmission electron microscopy (HRTEM) and STEM techniques. Figure 2(b) exhibits a high-angle annular dark-field (HAADF) STEM (Z-contrast) image of the YBCO layer on the STO substrate, which was taken when the electron beam was aligned with the 〈010〉 axis of STO. The image shows an atomic abrupt interface between the YBCO and STO. The orientation relationship between them can be determined as (001)_*Y BCO*_//(001)_*STO*_ and [010]_*Y BCO*_//[010]_*STO*_. Lattice bending and some defects were observed in the YBCO layers. Figure 2(c) exhibits LSMO, STO and YBCO layers. The thin (about 5 nm) STO layer is sandwiched between LSMO and YBCO. This STO layer has an orientation which is very similar to the substrate. The LSMO and STO layers have a cube to cube relationship. Most of lattice planes continue from YBCO to STO and LSMO. So these layers have an epitaxial growth. It should be noted that the interfaces in the multilayers are not flat at the atomic scale in Figure 2(c), which can be seen in Figure 2(a) as well.

**Figure 3:**
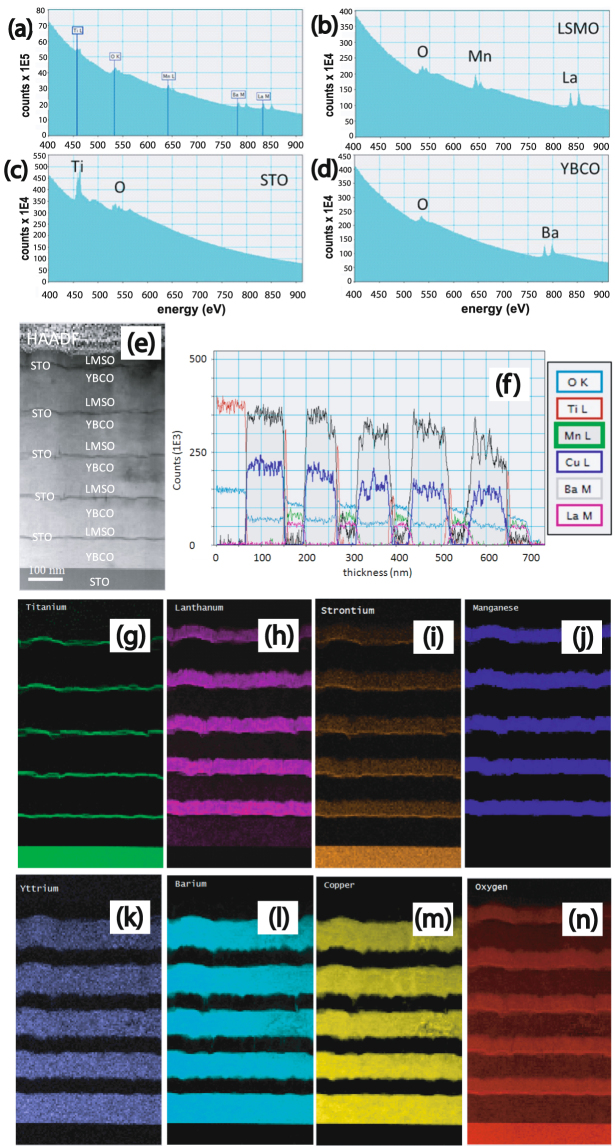
EELS measurements of specimen S2. (**a**) EELS spectrum in the energy-loss range of 400 eV–900 eV showing the Ti, O, Mn, Ba and La related energy loss edges of the specimen S2. (**b**–**d**) EELS spectra from LSMO, STO and YBCO. (**e**) Z-contrast image. (**f**) Plot of EELS elemental profiles within the sample. (**g**–**n**) Elemental EELS maps of Ti, La, Sr, Mn, Y, Ba, Cu and O.

Figure 3(a) displays an EELS spectrum in the energy-loss range of 400 eV–900 eV from the area which includes three different layers, where Ti, O, Mn, Ba and La related energy loss edges can be readily seen. Figure 3(b–d) display the EELS spectra from LSMO, STO and YBCO, respectively. Since Y, Cu and Sr do not have any energy-loss edges in 400 eV–900 eV, only O, Mn and La related energy loss edges show up in the LSMO spectrum (Fig. 3(b)). Similarly, Ti and O in STO (Fig. 3(c)) and O and Ba in the YBCO (Fig. 3(d)) spectrum are shown.

Elemental maps were obtained by using spectrum imaging technique. Besides Z-contrast image in Figure 3(e), Figure 3(f) shows the elemental profiles and Figure 3(g–n) show the elemental maps of Ti, La, Sr, Mn, Y, Ba, Cu and O. By comparing the maps, we may easily draw a conclusion that five LSMO/STO/YBCO trilayers were successively grown on the STO substrate. Figure 3(g) indicates that there are no pin holes across the SC-FM layers due to the thin STO layers. One may also note that the elemental profile of Cu does not show any signature of migration of Cu within LSMO. However, Figure 3(h,l) show some apparent intermixing of La into the YBCO and Ba into the LSMO which are due to the artifacts owing to the thickness effects on the deconvolution of the Ba and La edges. Thicker the sample, higher is the intensity of the post edge, which is often unrecognized by the software.

#### Ti chemical state

It was previously reported that Ti^3+^ ferromagnetism at heterostructural interfaces might result from charge transfer to the empty conduction band of STO and this charge transfer might tune magnetic alignment *via* double-exchange (DE) mechanism^13,14^. To verify this possibility we performed element specific EELS near edge structure measurements across the interface to isolate the interfacial region of interest and check whether there are any mixed valence states of Ti ions.

**Figure 4:**
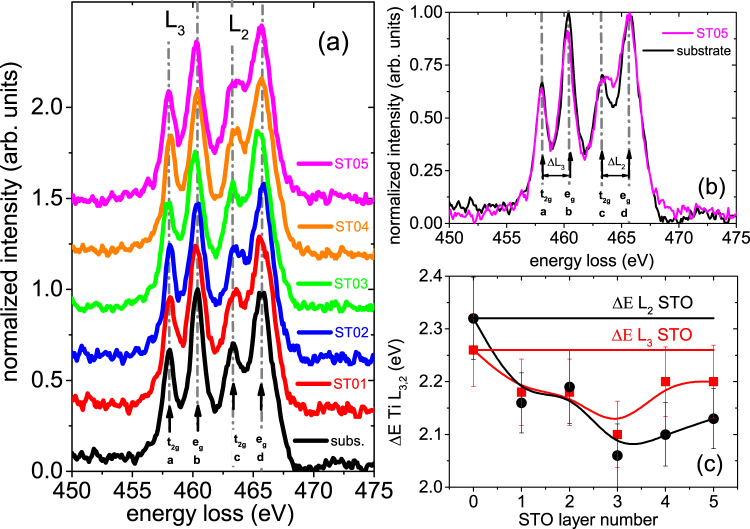
EELS L edge spectra of specimen S2. (**a**) EELS spectra in the energy-loss range of 450 eV–475 eV showing the Ti-edge of the specimen S2. Background subtraction and D-scan related energy shift correction were applied to all spectra. The plots have been normalized to L_2_ e_*g*_ peak height and have been vertically shifted for clarity. (**b**) Comparison of the Ti L-edge EELS spectra from STO5 and STO substrate without a vertical shift. (**c**) Energy difference between the e_*g*_ and t_2*g*_ peaks of the Ti L_3_ (red squares) and Ti L_2_ (black circles) absorption edges of the subsequent layers STO1–STO5 plotted as a function of the STO layer numbers (*n*=1,2...5) in the ML stack. The lines are a guide to the eye and the error bars are the experimental energy resolution of EELS. The horizontal lines are the ΔE L_3_ (red line) and ΔE L_2_ (black line) experimental values of the STO substrate of S2.

Figure 4(a) shows the EELS spectra of 5 STO layers (labeled as STO1–STO5 from bottom to top) in the range of 440 eV–470 eV. As a comparison, an EELS spectrum from the STO substrate is also included, where Ti exhibits the oxidation state of Ti^4+^. The Ti - L_3,2_ edge consists of four peaks, labelled as a, b, c, d. These peaks can be attributed to transitions from Ti 2*p* to Ti 3*d* levels with (a) 2p_3/2_ → 3*d*_*t*2*g*_, (b) 2p_3/2_ → 3*d*_*eg*_,(c) 2p_1/2_ → 3*d*_*t*2*g*_, (d) 2p_1/2_ → 3*d*_*eg*_. Usually, distinct stronger e_*g*_ peaks relative to the t_2*g*_ peaks at L_3,2_ edges signifies Ti^4+^ states^15^. It should be noted that to obtain these spectra, the original spectra were processed by background subtraction using a power law and re-plotted with an off-set considering D-scan related energy shift correction.

The overall shapes of the spectra across the Ti L_3,2_ edges are indicative of a mixed valence Ti^3+^ and Ti^4+^ states in the subsequent STO layers. Generally, Ti^3+^ (Ti^4+^) states have distinct characteristics where the t_2*g*_ (e_*g*_) peaks are relatively stronger than the e_*g*_ (t_2*g*_) peaks at the L_3_ (L_2_) edges. In our case, it is obvious that Ti ions do not have a pure Ti^3+^ state (Fig. 4(a)). The e_*g*_ and t_2*g*_ peaks of the STO layers are different from those of STO substrate. Ti edge signals of STO5 layer and STO substrate are compared in Figure 4(b), where a change in the relative strength is visible. This means that Ti ions do not have a pure Ti^4+^ state either.

In general, apart from the crystal field symmetry the crystal field strength can influence the details of the spectral shape. The e_*g*_ peak broadening is usually attributed to a minor distortion of the octahedral symmetry^16^ while t_2*g*_-e_*g*_ splitting and peak broadening are related to a drastic distortion of the TiO_6_ octahedra or a variation of the long range order^17^. The broadening of t_2*g*_ peaks can also be a sign of oxygen vacancies in STO^18^. The valence state of Ti ion in an oxide compound with mixed Ti^3+^ and Ti^4+^ ions may be determined quantitatively by the chemical shift of Ti L_3,2_ spectra^19^. Figure 4(c) shows the energy differences ΔE(L_3,2_) between between t_2*g*_ and e_*g*_ peaks at L_3_ and L_2_ edges associated with the STO layers^14^. The energy difference, which is related to the crystal-field splitting, indicate the presence of varying proportions of Ti^3+^ ions in the STO layers. The average Ti oxidation state can be calculated using the energy difference equation

1 $${\rm{\Delta }}E({L}_{\mathrm{3,2}})={\rm{\Delta }}E{({L}_{\mathrm{3,2}})}^{STO}-{\rm{\Delta }}E{({L}_{\mathrm{3,2}})}^{STOn}(\frac{{n}^{3+}}{{n}_{d}})$$

where *n*_*d*_ is the STO*n*(*n* = 1, 2,... 5) monolayer thickness and *n*^3+^ is the number of electron-doped monolayers. Electron doping of only half a unit cell is deduced from the values of ΔE(L_3_) and ΔE(L_2_) for STO*n* and STO substrate (horizontal lines in Figure 4(c)) using the above equation. Quantification of the electron doping in STO1-STO5 therefore yields *δ* = 0.5, approximately. Thus, we can confirm the existence of mixed valence state (Ti^(4+)−0.5^) where the electron residing in the t_2*g*_ orbitals of Ti band acts as an artificially doped titanate.

### X-ray absorption spectroscopy (XAS) measurements

#### Cu XAS

**Figure 5:**
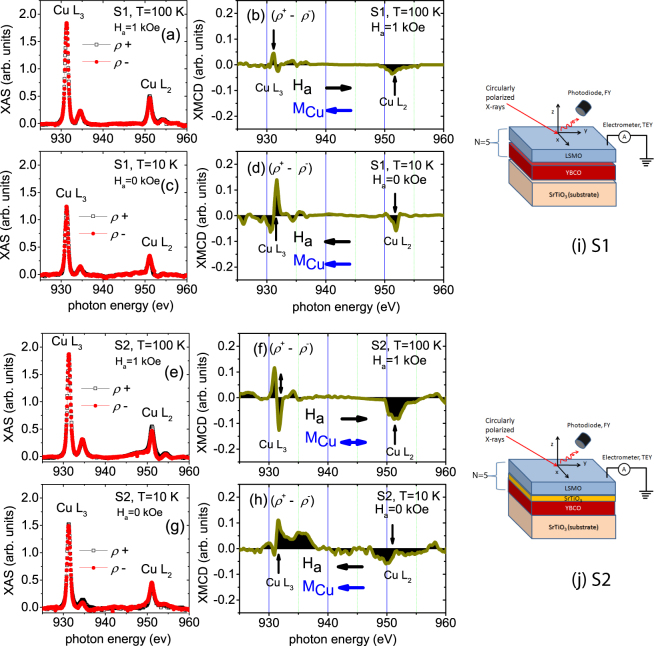
Cu L edge XAS and XMCD spectra of specimens S1 and S2. FY XAS with two different photon helicities (*ρ*^+^ and *ρ*^−^) and the corresponding XMCD signals of the Cu L_3,2_ edges from the S1 (**a**,**b**) and S2 (**e**,**f**) MLs at 100 K measured at 1 kOe and from the S1 (**c**,**d**) and S2 (**g**,**h**) MLs at 10 K measured at remanence after field cooling at 1 kOe. The XMCD signals have been multiplied by a factor of 10^2^. The XMCD signal at 100 K for the S2 ML is ambiguous. The horizontal arrows (black and blue) indicate the Cu magnetic moments direction with respect to the **H**_*a*_ direction. The vertical arrows indicate the positions of positive and negative XMCD signals of the L_3,2_ edges. Schematic pictures of the experimental configuration for (**i**) S1 and (**j**) S2 are also shown in the adjacent right panels.

Figure 5(a–f) show the fluorescence yield (FY) Cu L edge XAS spectra with different photon helicities (*ρ*^+^ and *ρ*^−^) and the corresponding XMCD spectra from the S1 and S2 MLs. The spectra of the Cu L_3,2_ edges were obtained in two different conditions of field and temperature, one at 100 K and 1 kOe (saturation field) (Fig. 5(a,b,e,f)) and the other at 10 K and 0 kOe (remanence) after cooling down the sample in 1 kOe (Fig. 5(c,d,g,h)). The experimental configurations for the MLs are schematically shown alongside.

Since the XMCD technique is only sensitive to the projection of the magnetic moments in the beam direction, it is not possible to establish whether the magnetic moments are aligned with the applied field or correspond to a non-collinear arrangement. As a general convention, it can be concluded that the magnetic moments are *parallel* to the applied field **H**_*a*_ where the dichroic signals are negative at L_3_ and positive at L_2_ edge. Similarly, the magnetic moments are *anti-parallel* to the applied field **H**_*a*_ where the dichroic signals are positive at L_3_ and negative at L_2_ edge. Note that the directions of **H**_*a*_ at saturation and at remanence after field cooling can be considered opposite to each other also by convention. This is in-spite of the fact that the magnetic moment direction at remanence is largely expected to retain the field cooling direction (Fig. 1(d)).

As the Cu XAS signal is one order of magnitude smaller than that of Mn, and as the Cu dichroism is another order of magnitude smaller, we had to average over many scans and do a careful smoothing to reduce the noise of the XMCD signal. The Cu L edge spectrum consists of two main peaks split by 20 eV corresponding to the L_3_ (931.3 eV) and L_2_ edge (951.3 eV), respectively. The extra multiplet features are due to different oxidization states of Cu within the YBCO layer. The first peak at 931.3 eV is due to the Cu^2+^/Cu^3+^ ions, the peak at 934.5 eV is due to the Cu^1+^ ions which can be associated with oxygen deficiency^20^. Similarly, a huge difference in the spectra between 10 K and 100 K in the range of 934 eV–938 eV can be seen, most significantly for S2. The strong multiplet effects restrict the detailed analysis of spin and orbital moments.

In Figure 5(b,d,h), the opposite signs of the XMCD signals at the L_3_ and L_2_ edges provide the evidence of induced magnetism of Cu at 100 K (above *T*_SC_ ≈ 50 K) in S1 and also at 10 K (below *T*_SC_) in both samples. The magnetic origin of Cu is further confirmed as these signals are compared with the non-magnetic signal which is positive at both L_3_ and L_2_ edges measured at 300 K and 1 kOe (see Supplementary Information: Fig. 1)^10^. As expected, we do not see a reversal of the dichroic signals for S1 between at 100 K and 1 kOe (Fig. 5(b)) and at 10 K and remanence (Fig. 5(d)) even though there is a change in the applied field direction. This signifies that the Cu magnetic moments are anti-parallel to the **H**_*a*_ direction (the dichroic signals are positive at L_3_ and negative at L_2_ edge) at 100 K and 1 kOe while they are considered to be parallel at 10 K and remanence.

It should be noted that the Cu signals obtained in FY mode are associated with the average response of all Cu atoms in the whole cuprate structure of the 30.0 nm-thick of YBCO layer. Since there is a 30.0 nm LSMO layer on top of the YBCO layer, the Cu XMCD signal is too weak (only 3% at 100 K and 12% at 10 K of the total signal) to provide further information about Cu magnetism in the present case. The Cu XMCD signal is even weaker in S2 due to the intervening STO layer. Nevertheless, the signature of Cu magnetism is evident, at least at 10 K and remanence for S2 (Fig. 5(h)). The Cu magnetic moments indicate an orientation similar to that of S1 at the same temperature and field conditions. The XMCD signal at 100 K (Fig. 5(f)) is ambiguous for S2 and can be interpreted as unclear magnetism, similar to that reported earlier in a similar system^21^. It was reported that at 100 K, there was no Cu magnetic moment for a 25 u.c. YBCO layer in the LCMO/YBCO system^9^, where *T*_SC_ was 80 K which is slightly higher than in our system.

For S1, a 23% decrease in the Cu XMCD signal is observed when the temperature increases from 10 K to 100 K and can be attributed to a competition between the thermal disorder and the magnetic field in the system^22^. This temperature dependence is in agreement with the earlier observations in similar YBCO/LCMO systems^3,10^. For S2, since the signal at 10 K is even lower than for S1, such thermal effect on the signal becomes irrelevant. The presence of Cu magnetism below their *T*_SC_s in S1 and S2, with or without the STO intervening layer, is very interesting from the point of view of superconductivity as it confirms simultaneous presence of superconductivity and intrinsic magnetic moments of Cu.

#### Mn XAS

**Figure 6:**
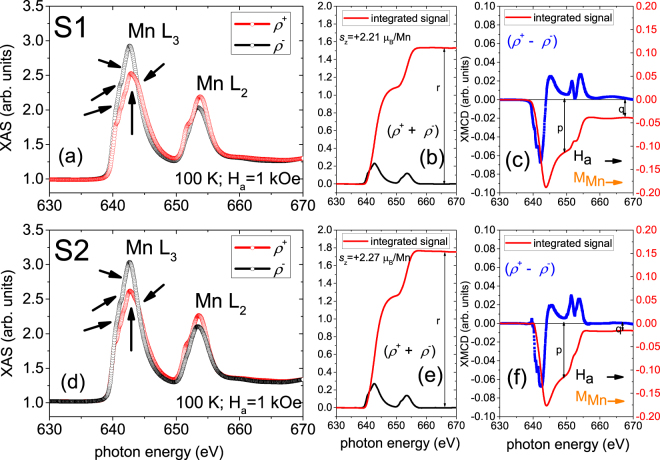
Mn L edge XAS, XMCD spectra and sum rules for *s*_*z*_ of specimens S1 and S2. TEY XAS signals of the Mn L_3,2_ edges with two different photon helicities (*ρ*^+^ and *ρ*^−^) from the (**a**) S1 and (**d**) S2 MLs measured at 100 K and 1 kOe (saturation). XAS (summation) and XMCD (difference) signals corresponding to the XAS signals for S1 (**b**,**c**) and S2 (**e**,**f**) after baseline correction that takes into account of the linear increase of the background. The p, q and r values are indicated inside. The red lines are the integrated area of each signal. The arrows (black and orange) indicate the Mn magnetic moments direction with respect to the **H**_*a*_ direction.

Figure 6(a–f) shows the total electron yield (TEY) Mn L_3,2_ edges XAS spectra with different photon helicities (*ρ*^+^ and *ρ*^−^) and the corresponding XMCD signals obtained at 100 K and 1 kOe (saturation). Figure 6(a–c) show the signals for S1 and in Figure 6(d–f) for S2. Figure 6(b,c,e,f) display the corresponding integrated area under the XAS (summation: *ρ*^+^ + *ρ*^−^) and XMCD (difference: *ρ*^+^ − *ρ*^−^) curves. Sum rules, described in the methods section, give the spin component of magnetic moment *s*_*z*_ = +2.21 ± 0.05 *μ*_*B*_ per Mn atom (and the orbital component *l*_*z*_ = 0.19 ± 0.05 *μ*_*B*_ per Mn atom) in S1 and +2.27 ± 0.05 *μ*_*B*_ per Mn atom (*l*_*z*_ = 0.07 ± 0.05 *μ*_*B*_ per Mn atom) in S2. Note that the signs of the Mn dichroism signals with respect to the **H**_*a*_ direction (the dichroic signals are negative at L_3_ and positive at L_2_ edges) indicate an alignment of the Mn magnetic moments with the **H**_*a*_ direction for both MLs, S1 and S2. Comparing the dichroism signals of Mn (Fig. 6(c)) and Cu (Fig. 5(b)), both measured at 100 K (above *T*_SC_ ≈ 60 K) and saturation, an anti-parallel coupling between LSMO and the YBCO is revealed across the hybrid heterostructure interfaces of S1. For S2, no such coupling could be established at 100 K due to unclear Cu magnetism.

The Mn L edge splits into two separate multiplets L_3_ (at 642 eV) and L_2_ (at 653 eV) due to spin-orbit interaction of the core states. The XAS L_3,2_ edges show no double peak structures but narrow peak width with shoulders. This strongly suggests the absence of MnO like contribution^23^. The Mn L_3_ peak located at around 642.4 eV indicates that the main valence state is Mn^3+^. Four shoulders can be identified at 640.5 eV, 641.1 eV, 641.8 and 643.6 eV which can be attributed to Mn, Mn^2+^, Mn^2+/3+^ and Mn^4+^, respectively (see Supplementary Information: Fig. 2). Therefore, these results indicate the coexistence of Mn^2+^, Mn^3+^ and Mn^4+^ in both samples which can be attributed to the inhomogeneous distribution of ${{\rm{O}}}_{2}^{-}$ ions^10^.

**Figure 7:**
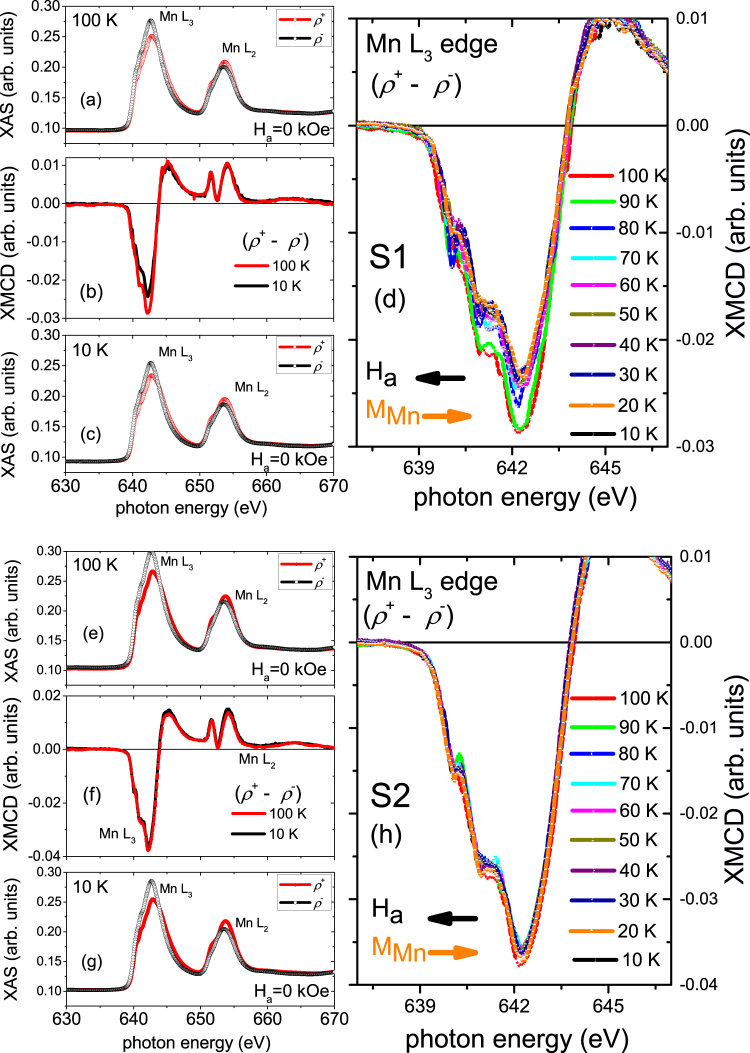
Mn L edge XAS and XMCD spectra of specimens S1 and S2. TEY XAS with two different photon helicities (*ρ*^+^ and *ρ*^−^) and the corresponding XMCD signals of the Mn L_3,2_ edges measured at remanence after field cooling at 1 kOe from the S1 (**a**–**d**) and S2 (**e**–**h**) MLs. The XAS measurements (**a**,**e**) at 100 K and (**c**,**g**) at 10 K are shown in two separate panels while the XMCD data are plotted together in the middle panels (b,f) for S1 and S2. Temperature dependence of the L_3_ edge XMCD signals are shown for S1 (**d**) and for S2 (**h**). The arrows (black and orange) indicate the Mn magnetic moments direction with respect to the **H**_*a*_ direction. The maximum dichroic signal is ≈62% at high T and decreases significantly to ≈50% as T crosses below *T*_SC_ for S1. The maximum dichroic signal is ≈22% at high T and decreases nominally to ≈20% as T crosses below *T*_SC_ for S2. The percentages are calculated with respect to the XMCD signal at 10 K from a reference sample of LSMO layer on STO without a YBCO layer.

Even though the TEY mode probes the top 5.0 nm Mn layer which is near the ambient/LSMO interface of the MLs, proximity effect is still expected as the Cooper pairs were reported to affect the entire 30.0 nm-thick ferromagnetic layer^24^. The Mn L_3,2_ edges of S1 and S2 were further investigated as a function of temperature, traversing through their *T*_SC_s. Figure 7(a–h) show the TEY Mn L_3,2_ edge XAS obtained with different photon helicities (*ρ*^+^ and *ρ*^−^) and the corresponding XMCD signals obtained for S1 (Fig. 7(a–d)) and for S2 (Fig. 7(e–h)), respectively. The measurements were done at various temperatures between 100 K and 10 K and at remanence after cooling down the sample in 1 kOe. Here, at remanence, the signs of Mn dichroism signals (the dichroic signals are negative at L_3_ and positive at L_2_ edges) indicate an anti-parallel alignment of the Mn magnetic moments with the **H**_*a*_ direction for both MLs, S1 and S2. The opposite signs of the Mn (Fig. 7(b,f)) and Cu (Fig. 5(d,h)) dichroism signals, both measured at 10 K (below *T*_SC_) and remanence, reveal an anti-parallel coupling between LSMO and YBCO across the hybrid heterostructure interfaces of S1 and S2 MLs.

For S1, one may note the obvious difference in the Mn XMCD signals of the L_3_ edge (642.2 eV) obtained at 100 K (above *T*_SC_) and 10 K (below *T*_SC_), which is shown in Figure 7(b). These signal intensities are similar to that reported on similar systems^10^. Figure 7(d) shows the temperature dependence of the L_3_ edge XMCD signals for S1, thus revealing sudden changes in them as the temperature traverses through *T*_SC_. The XMCD signal (Mn magnetic moments) continue to decrease until the temperature drops below *T*_SC_. This decrease cannot be due to the flux expulsion by the Meissner effect in the superconducting layer^25^, but is due to the formation of smaller domains^26^ which are commonly observed in YBCO/LCMO superlattices^8^.

For S2, the Mn XMCD signals of the L_3_ edge were measured under the same conditions as S1. They were obtained above *T*_SC_ at 100 K and below *T*_SC_ at 10 K (Fig. 7(f)). Here, the difference in the XMCD signals is not that obvious as it was for S1. The temperature dependence of the L_3_ edge XMCD signals is shown in Figure 7(h) where their differences with temperature are more clear. Following the same sum rules mentioned in the methods section, the spin magnetic moment s_*z*_ value of Mn at 10 K is calculated to be +1.68 ± 0.05 *μ*_*B*_ per Mn atom (*l*_*z*_ = 0.38 ± 0.05 *μ*_*B*_ per Mn atom) for S2. This magnetic moment value is smaller than that in bulk (3.33 *μ*_*B*_ per Mn atom)^27^. One possible explanation can be that the magnetic moments in the bottom part of the Mn layer is smaller than that in the upper part due to a possible dead layer formation.

**Figure 8:**
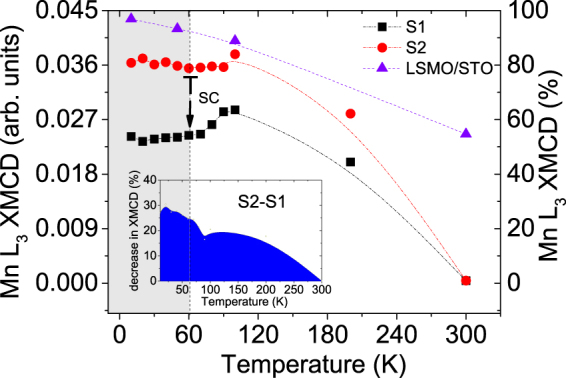
XMCD at the Mn L_3_ edge of specimens S1 and S2. The temperature dependence of XMCD peak heights of the Mn L_3_ edge from S1 (black square) and S2 (red circle). The dotted line indicates *T*_SC_ as obtained from the magnetization data. The error bars are typically the symbol sizes. The data has been compared with a reference Mn signal (violet triangle) from a LSMO layer not interfaced with YBCO. Inset shows the percentage difference in XMCD signal with temperature, normalized to the signal from a LSMO reference sample, indicating a 30% loss of magnetization in S1 with respect to S2 at 10 K.

Figure 8 further depicts the temperature dependence of the XMCD peak heights of the Mn L_3_ edge for both S1 and S2 in an extended temperature range above and below *T*_SC_. These data sets have been normalized to the XMCD peak height measured at 10 K and remanence from a reference sample with a layer of LSMO on STO (001) substrate, which has no superconducting layer adjacent to the LSMO interface. Far above *T*_SC_, the Mn L_3_ edge peak height reduces drastically, which is in accordance with our previous FC SQUID data (Fig. 1(b)). The multiplet broadening of the L_3_ peak mentioned earlier, is a consequence of partial occupation of the Mn *d* orbitals. The dichroism peak height of the L_3_ edge can be used as the signature of percentage change in Mn magnetism with temperature^10^. Below *T*_SC_, the systematic loss of the XMCD peak height of S1 as compared to S2 (shown as a percentage change in the inset) is in accordance with the typically observed proximity-induced loss of ferromagnetic ordering in LSMO. The strong suppression of magnetism in S1 which is 50% as compared with the reference sample, is therefore due to the usual proximity effect, where the Cooper pairs could affect the entire ferromagnetic layer on top. In fact, the inner LSMO/YBCO layers of the ML stack should also be affected since a modulation of the FM magnetization within a ML stack was reported earlier by Hoppler *et al*.^24^. Interestingly, a decrease of the Mn magnetism can be seen at 10 K in S2 as well. However, in S2, the proximity effect is reduced at least by 20%. Without the tunneling of Cooper pairs across STO, the Mn XMCD signal would have been much stronger^6^. This is also evident from the fact that there is a loss of Mn magnetization by 30% in S1 as compared to S2 (inset of Fig. 8).

### Polarized neutron reflectivity (PNR)

**Figure 9:**
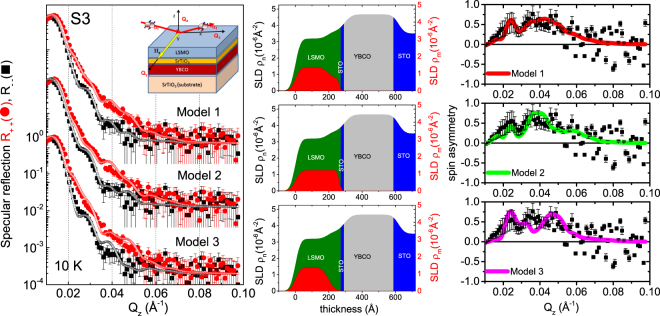
PNR measurements of specimen S3. Specular neutron reflectivity patterns (solid symbols) along with their best fits (open symbols) as a function of *Q*_z_ for the NSF [R_−−_ (black) and R_++_ (red)] channels measured at the saturation field **H**_*a*_ = +1.0 kOe and 10 K for the trilayer sample S3 using model 1, model 2 and model 3. Inset shows a schematic of the magnetic field measurement and neutron scattering geometry. The nuclear (*ρ*_*n*_) and magnetic (*ρ*_*m*_) SLDs versus the thickness of the trilayer are shown alongside. Also shown are the spin asymmetry data and the corresponding fits using the three different models, model 1, model 2 and model 3.

It was reported that the average in-plane FM ordering of the surface could be significantly suppressed over a length scale of about 4 u.c. from the surface, even at low temperature^28^. Thus the reduced Mn magnetization was further investigated using a depth sensitive technique like polarized neutron reflectivity (PNR). Figure 9 shows the polarized neutron intensity profiles of the trilayer sample S3 along *Q*_z_ and their fitting curves after the sample was cooled down in a saturating field of +1.0 kOe and measured at +1.0 kOe at 100 K and at 10 K. S3 with a single LSMO layer was chosen to avoid the complexity caused by a multilayer sample. For a multilayer, it would be difficult to resolve magnetic variation within the different LSMO layers^29^. The fitting curves were done using simple models of block-potentials. The parameters that were used for fitting are the individual layer thicknesses, the nuclear (*ρ*_*n*_) and and magnetic (*ρ*_*m*_) scattering length densities (SLDs) of the individual layers. The *ρ*_*n*_ and *ρ*_*m*_ values are shown alongside in Figure 9. The errors in the thickness of the layers are typically ±0.2 nm, while that for the nuclear and magnetic scattering length densities *ρ*_*n*_ and *ρ*_*m*_ values are ±0.1 × 10^−6^ Å^−2^ and ±0.05 × 10^−6^ Å^−2^, respectively. The interface roughness is ≈2.0 ± 0.5 nm. Fitted parameters were obtained using the minimization of chi squared (*χ*^2^ or the goodness of fit) value.

The PNR spectra measured at 10 K show the splitting of the R_++_ and R_−−_ profiles, a signature of net magnetization within the sample. Depth sensitivity of PNR allows us to deduce the nuclear and magnetic SLD profiles throughout the entire thickness of the film from the fits. We have considered three possible models (model 1, model 2 and model 3) for the purpose. The models explore the existence of a possible magnetic dead layer at the interface. Model 1 assumes that a magnetization at the interface of LSMO-STO is extended within one third of the total thickness of LSMO and the interface area has a magnetic moment value lower than the rest of the LSMO layer. Model 2 assumes that the magnetization is uniform throughout the entire thickness of the LSMO layer. Model 3 assumes a magnetic dead layer at the STO interface. A plot of the spin asymmetry of the profiles [(R_++_ − R_−−_)/(R_++_ + R_−−_)] along with the fits corresponding to the three models are also shown alongside in Figure 9. The *χ*^2^ value for model 1 is slightly better than model 2 and worsens for model 3. Thus, we cannot ascertain a complete dead layer formation^30^. A reduced magnetic moment value at the interface seems to be more plausible here. Nonetheless, all models yield a reduced average magnetic moment value of around +2.25 ± 0.05 *μ*_*B*_ per Mn atom which is significantly lower than in bulk or thin film of LSMO on (001) STO (3.33 *μ*_*B*_ per Mn atom)^27^. Thus the PNR profile analysis supports our XMCD results at 10 K. The PNR profiles measured at 100 K (see Supplementary Information: Fig. 3) could not confirm any anomaly of the Mn magnetic moment value around *T*_SC_.

## Discussions

For S1, the Cu and Mn dichroic signals measured at 100 K (above *T*_SC_) and 1 kOe show that the Cu and Mn magnetic moments are anti-parallel to each other. When measured at 10 K (below *T*_SC_) and remanence, the Cu and Mn magnetic moments continue to remain anti-parallel both for S1 and S2. Thus for S1, the Cu and Mn magnetic moments are anti-parallel to each other below and above *T*_SC_. For S2, one may recall that the Cu dichroism is ambiguous at 100 K as the magnetic moments are either not induced above *T*_SC_ or are unclear. Furthermore, the Mn or Cu dichroic signals at remanence do not show any change in their signs with temperature. Previously, the antiferromagnetically coupled Mn and Cu XMCD signals were shown to decrease monotonically across *T*_SC_ as a function of temperature when measured at saturation in similar systems^8,10^.

The presence of Cu within LSMO could have been a clinching evidence of quantum mechanical tunneling of Cooper pairs across an insulator. The anti-parallel coupling of Mn-Cu could also serve as an indirect evidence of quantum mechanical tunneling of Cooper pairs which also induces lower magnetization on the ferromagnet side. Lowering of ferromagnetic magnetization was reported earlier in YBCO/STO/LCMO system as well^12^. However, recent report on YBCO/LCMO system suggests that Cu magnetic moments reside on the YBCO side, thus ruling out the possibility of migration of Cu atoms into the LCMO layer^31^. Absence of Cu migration is also evident from the elemental EELS profiles in our YBCO/STO/LSMO samples. Nonetheless, due to the presence of intervening STO in our system, the mechanism of antiferromagnetic coupling between Mn and induced Cu magnetic moments may require a different explanation.

The Mn ions in our samples are in a mixed valence state. It has been reported earlier that Mn^3+^ valence state has a reconstructed e_*g*_ orbital, preferentially occupying the ${d}_{3{z}^{2}-{r}^{2}}$ or ${d}_{{x}^{2}-{y}^{2}}$ orbitals, due to a compressive or tensile strain in the *a*–*b* plane, respectively. The magnetic behavior is thereby influenced by strong spin-lattice coupling along the in-plane direction^32^. In a typical cuprate-manganite interface (sample S1, for example), the antibonding ${d}_{3{z}^{2}-{r}^{2}}$ orbitals have an energy higher than the Cu ${d}_{{x}^{2}-{y}^{2}}$ orbitals. This lowering of energy allows the transfer of electrons from Mn to Cu ${d}_{{x}^{2}-{y}^{2}}$ bands with anti-parallel spin^3^. The magnetic moments of Cu ions was induced at the interface by the presence of the neighboring Mn spins *via* Cu-O-Mn covalent bonding. Recently, it has been established that even in the absence of a covalent bonding, the interfacial Cu_2_O planes can develop weak ferromagnetic behavior associated with the transfer of spin-polarized electrons from the LSMO layer^21^.

In cuprate-titanate-manganate interface, the situation is different. Our high resolution Ti EELS spectra indicate that there are Ti^(4+)−*δ*^ states in the five intervening STO layers *i.e*., Ti exists in a mixed valence (3^+^/4^+^) state. The 2*p* orbitals of the O_2_ atoms in the STO layer act as an intermediate medium of electron transfers forming the orbital couplings between (i) Mn 3*d* - O 2*p* - Ti 3*d* (Mn^3+^ - Ti^3+^) with antiferromagnetic coupling *via* superexchange (SE) interaction mechanism at one interface of STO and (ii) Ti 3*d* - O 2*p* - Cu 3*d* (Ti^3+^ - Cu^2+^) orbitals with ferromagnetic coupling *via* double-exchange (DE) interaction mechanism at the other interface of STO.

Depending upon the strain state of the superlattice, the sign of the spin-spin coupling at the interface can alter from antiferromagnetic to ferromagnetic which essentially outlines the role of orbital reconstruction^14^. While the overlap of the t_2*g*_ bands of Mn and Ti is relatively small, an orbital hybridization may result from the preferentially occupied Mn ${d}_{3{z}^{2}-{r}^{2}}$ and empty Ti ${d}_{3{z}^{2}-{r}^{2}}$ bands. Since t_2*g*_ orbitals in titanates are located near the Fermi level of manganite, one may need to consider t_2*g*_ orbitals carefully^33^. According to Goodenough-Kanamori rules, the dominant SE process between the Ti t_2*g*_ electrons (*d*_*xz*_ and *d*_*yz*_) and Mn ${d}_{3{z}^{2}-{r}^{2}}$ bands is antiferromagnetic.

**Figure 10:**
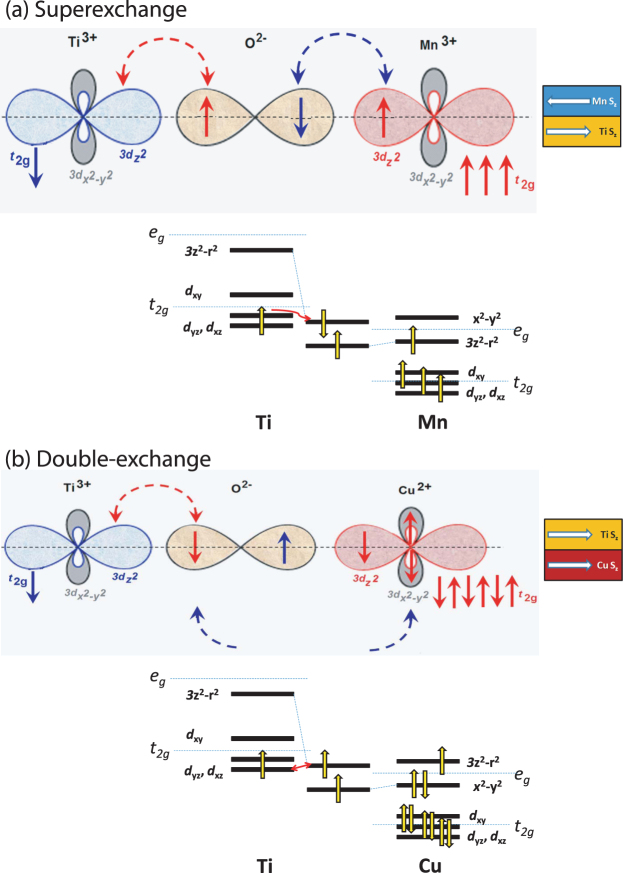
SE and DE coupling mechanisms. Schematic diagram of (**a**) Superexchange interaction between Mn 3*d*-O 2*p*-Ti 3*d* (Mn^3+^-Ti^3+^) and (**b**) Double-exchange coupling between Ti 3*d*-O 2*p*-Cu 3*d* (Ti^3+^-Cu^2+^) orbitals in the hybridization process. In one case 3*d*(3*z*^2^ − *r*^2^) orbital is occupied (LSMO), whereas in the other case the 3*d*(3*z*^2^ − *r*^2^) orbital with higher energy and 3*d*(*x*^2^ − *y*^2^) orbital with lower energy are occupied.

The above scenario can be quite different in the case of Cu where the Cu ${d}_{3{z}^{2}-{r}^{2}}$ bands remain partially occupied. The ${d}_{{x}^{2}-{y}^{2}}$ bands are at a lower energy level and their position is nearly identical to the occupied band of manganites (e.g. LSMO). Therefore, the charge transfer from Ti t_2*g*_ to Cu ${d}_{{x}^{2}-{y}^{2}}$ can easily occur. The ${d}_{{x}^{2}-{y}^{2}}$ orbital becomes energetically favorable for the electrons. The hole density in Cu is thus transferred to the ${d}_{3{z}^{2}-{r}^{2}}$ orbital. Transfer of charge occurs simultaneously as one electron is transferred from the bridged oxide to the empty ${d}_{3{z}^{2}-{r}^{2}}$ bands of Ti and is replaced by another from the ${d}_{3{z}^{2}-{r}^{2}}$ bands of Cu. Thus ferromagnetic Hund coupling is facilitated here *via* DE process. Schematic diagrams of the hybridization processes *via* SE and DE mechanisms are illustrated in Figure 10. The overall coupling between Mn 3*d* - Cu 3*d* therefore remains antiferromagnetic even through the STO layer.

## Conclusions

We have grown two MLs of YBCO/LSMO and YBCO/STO/LSMO and a trilayer of YBCO/STO/LSMO superlattices on STO (001). The MLs were studied by circularly polarized X-ray absorption at the Cu and Mn L_3,2_ edges and layer specific EELS. The trilayer was exclusively studied by PNR. The main conclusions of this work can be summarized as follows.

(i) From the XMCD signals we observed induced Cu magnetic moments, anti-parallel to Mn magnetic moments below *T*_SC_, for both multilayers. Close to *T*_SC_, the Mn magnetization in the YBCO/LSMO ML was seen to be significantly reduced by around 50% as compared to the that in a single LSMO layer grown on STO (001). This reduction of magnetization is a clear evidence of the *proximity* effect. Interestingly, we observed a significant decrease of the Mn magnetization at the interface of the YBCO/STO/LSMO ML as well. However, here, the effect was reduced by 20%.
(ii) Furthermore, depth sensitive PNR profiles were used to determine the magnetic profile of the YBCO/STO/LSMO trilayer. The results confirmed the reduction of Mn magnetic moment value at the interface of the trilayer when measured at 10 K (below *T*_SC_). Thus the XMCD results were corroborated by the PNR profile analysis.
(iii) Finally, based upon the EELS spectra, we observed a strong indication of Ti^(4+)−*δ*^ states form the STO layers with the YBCO/STO/LSMO ML. This indicates that the electrons can migrate from Ti^(4+)−*δ*^ to Mn^3+^ and from Ti^(4+)−*δ*^ to Cu^2+^ with band hybridization *via* O 2*p* orbitals.

The decrease in the suppression of Mn magnetic moments (from 50% to 20%) and the induced anti-parallel Cu moments in the YBCO/STO/LSMO ML indicate either a tunneling and/or a transfer of charge that can take place across the insulating STO layer. Owing to the presence of Ti^(4+)−*δ*^ states, the coupling below *T*_SC_ is explained by a combination of SE and DE mechanisms across the STO interface on either side of the YBCO and LSMO layers. Thus, the responsible mechanism for the reduced proximity effect in the YBCO/STO/LSMO ML is owed to the charge-transfer driven orbital ordering.

This novel interface magnetism is a big step forward in exploring the fundamental aspect of superconducting order parameter via long range proximity effect and consequent coupled magnetic modulation in these hybrid heterostructures. The magnetic-bridge across an insulating layer like SrTiO_3_, can be seen as a general but novel aspect which can be exploited in cases involving SC-FM spin valves.

## Methods

### X-ray diffraction and magnetization measurements

The degree of crystallinity of the heterostructures was evaluated by X-ray diffraction (XRD) measurements and the layer thicknesses were determined from X-ray reflectivity (XRR). The X-ray measurements were carried out using Cu K_*α*_ radiation from a rotating anode X-ray source. Conventional in-plane magnetization loops were measured at various temperatures and fields using a superconducting quantum interference device (SQUID) from Quantum Design.

### TEM measurements

Tescan GAIA3 SEM/FIB, Fischione Nanomill and SBT IBS/e sputter coater were used in cross-sectional transmission electron microscopy (TEM) specimen preparation. JEOL JEM-2100 TEM was used for conventional TEM imaging, HRTEM imaging and electron diffraction. JEOL Grand ARM equipped with Gatan GIF Quantum was used to obtain scanning TEM (STEM) and element specific electron EELS data at 200 kV. Gatan DitalMicrograph (GMS) software was used for data analysis. The cross-sectional TEM specimens were prepared by using a focus ion beam in a double beam system. The ML sample S2 was first coated with a thin carbon layer and then protected by Pt layers before ion beam treatment of the sample. Routine procedures were used to cut and thin the specimen, and the sample was finally polished with a low Ar^+^ ion beam at 300 eV in Fischione Nanomill to get rid of Ga implantation and reduce amorphous layers.

### X-ray absorption measurements

X-ray absorption spectroscopy (XAS) and X-ray magnetic circular dichroism (XMCD) measurements were done as function of photon energy (E) by using the depth sensitive Fluorescent yield (FY) photons for the Cu edge and total electron yield (TEY) for the Mn edge at various temperatures at the beamlines UE56/2-PGM1 of BESSY II, Helmholtz Center Berlin. We have utilized the different probing depths of different detection modes (TEY with probing depth of 5 nm and FY, with probing depth of 100 nm). The respective depth limitations restrict us to investigate mostly the first LSMO and YBCO layers on the top of the ML stack.

We probe across the Cu and Mn L_3,2_ edges for their magnetic and orbital properties at the interface. The spectra *ρ*^+^ and *ρ*^−^ represent the parallel and anti-parallel alignment of the magnetization direction with the photon helicity vector. XMCD signal is angle dependent and is proportional to k · M, where k is the X-ray propagation vector and M the magnetization vector. The angle *θ* between M and k was set at 10 in order to observe the in-plane magnetic signal. The L edge (resulting from the 2*p* to 3*d* dipole transitions) gives L_3_ (2*p* ${\textstyle \tfrac{3}{2}}$) and L_2_ (2*p* ${\textstyle \tfrac{1}{2}}$) edges in the spectra. These peaks can be attributed to transitions from 2*p* to 3*d* levels with 2*p*_3/2_ → 3*d* and 2*p*_1/2_ → 3*d*. The spectra are sensitive to factors that change 3*d* orbital splitting and occupation, such as spin configuration, ligand field and manganese valence. The XAS is the summation and XMCD is the difference of *ρ*^+^ and *ρ*^−^. The degree of polarization was nearly 95%. The samples were measured either at saturation or at remanence after applying a field of around 1 kOe (applied along the direction of the incident X-rays). This means that the Mn magnetization does not change sign at remanence but only reduces in magnitude by few percentage from saturation. The right-handed and left-handed circularly polarized XAS spectra were obtained by reversing the photon helicity. In principle it is possible to use sum rules to get an estimate of the effective spin (*s*_*z*_) and orbital (*l*_*z*_) components of the magnetic moment in saturation and for cubic symmetry (neglecting the dipole component). The t_*z*_ dipole term for transition metal in cubic crystal field is expected to be zero from symmetry considerations and can be neglected compared to S_*z*_ in the spin sum rule. The magnetic moments can be obtained from the following equations:

2 $${s}_{z}=-\frac{\mathrm{(6}p-4q)}{r}{n}_{h}$$

3 $${l}_{z}=-\frac{4q}{3r}{n}_{h}$$

where n_*h*_ is the number of holes in *d* shell, r is the average of the integrated area of the absorption spectra $({\rm{r}}={\int }_{{L}_{3}+{L}_{2}}\,({\rho }^{+}+{\rho }^{-}){\rm{dE}})$ for the two circular polarizations, q $({\rm{q}}={\int }_{{L}_{3}+{L}_{2}}\,({\rho }^{+}-{\rho }^{-}){\rm{dE}})$ and p $({\rm{p}}\,=\,$ ${\int }_{{L}_{3}}\,({\rho }^{+}-{\rho }^{-}){\rm{dE}})$ are the values of the integrated XMCD signal after the L_3,2_ edges and between the L_3_ L_2_ edges, respectively. The nominal composition of the LSMO layers corresponds to a density of n_*h*_ = 6.33 holes or n_*e*_ = 3.67 electrons per Mn ion in the 3*d* band.

### Polarized neutron reflectivity

Polarized neutron reflectivity (PNR) measurements for the samples were performed at the neutron reflectometer MARIA at FRM II, Germany. The instrument was used at a wavelength *λ* = 4.5 Å with a focused beam on the 1 cm^2^ sample size. The beam is polarized by a polarizing guide and analyzed by a wide angle ^3^He-cell^34^. An in-plane magnetic field of 1 kOe was used to saturate the LSMO layer before the samples were cooled in a closed-cycle cryostat.

From the neutron polarization analysis we resolve the different components of the magnetization within the film plane as only the magnetic moments within the sample plane contributes to the scattering. The scattering length densities (SLD) of a specimen are given by the nuclear (*ρ*_n_) and magnetic (*ρ*_m_) components of the SLD. Two different cross sections were measured namely, the non-spin flip (NSF) channels represented by R_++_ and R_−−_. Here + and − signs are used to distinguish the intensity contributions *R* representing a polarization component parallel or anti-parallel to the guiding field, respectively. The NSF scattering amplitude provides information about *ρ*_n_ ± *ρ*_m_ cos *ϕ*_A_. We designate *ϕ*_A_ as the angle between the direction of FM magnetization (**M**_*FM*_) and the neutron spin quantization axis. The neutron polarization vector is guided by the field applied to the sample (**H**_*a*_) along the y-axis. Since we have measured in saturation, *ϕ*_A_ = 0.

## Electronic supplementary material

Supplementary Information
