# Supplementary material for: Proximity effects across oxide-interfaces of superconductor-insulator-ferromagnet hybrid heterostructure
Source: Sci Rep. 2018 Feb 27;8:3732. doi: 10.1038/s41598-018-22036-y (PMC5829237; doi:10.1038/s41598-018-22036-y)
Supplement: Supplementary file 1 — Supplementary Information [file 41598_2018_22036_MOESM1_ESM.pdf]

Supplementary Information: Proximity effects across oxide-interfaces of superconductor-insulator-ferromagnet hybrid heterostructure

C. L. Prajapat[1], Surendra Singh[2,3], D. Bhattacharya[2], G. Ravikumar[1], S. Basu[2,3], S. Mattauch[4], J-G. Zheng[5], T. Aoki[5], and Amitesh Paul[6*]

[1] *Technical Physics Division, Bhabha Atomic Research Centre, Mumbai-400085, India*

[2] *Solid State Physics Division, Bhabha Atomic Research Centre, Mumbai-400085, India*

[3] *Homi Bhabha National Institute, Anushaktinagar, Mumbai 400085, India*

[4] *Jülich Centre for Neutron Science (JCNS) at Heinz Maier-Leibnitz Zentrum (MLZ), Forschungszentrum Jülich GmbH, Lichtenbergstraße 1, D-85747 Garching b. München, Germany*

[5] *Irvine Materials Research Institute, University of California, Irvine, CA 92697-2800, USA and*

[6] *Technische Universität München, Physik Department E21, Lehrstuhl für Neutronenstreuung, James-Franck-Straße 1, D-85748 Garching, Germany*

PACS numbers: 75.70.Cn, 61.05.fj

*Corresponding author; Electronic address: `amitesh.paul@tum.de`

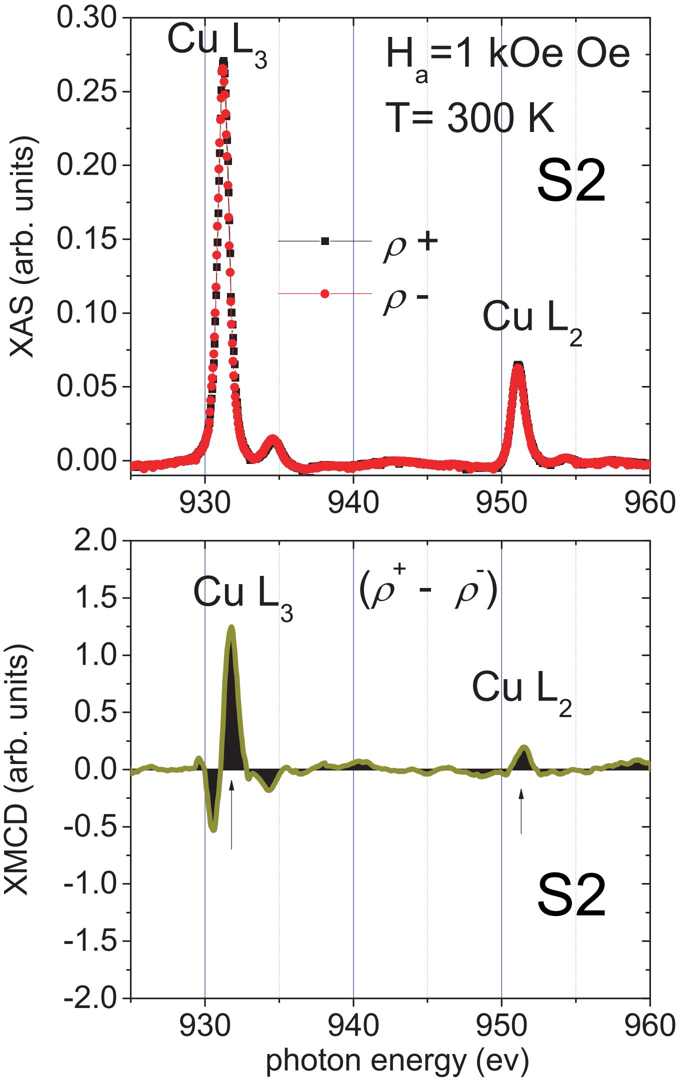

FIG. 1: (Color online) **Cu L edge XAS and XMCD spectrum of specimen S2 at 300 K.** FY XAS for the Cu $L_{3,2}$ edges with two different photon helicities (ρ^+ and ρ^-) and the corresponding XMCD measurements from the S2 ML measured at 300 K and 1 kOe. The XMCD signals have been multiplied by a factor of 10^2. The signal indicates a non-magnetic Cu.

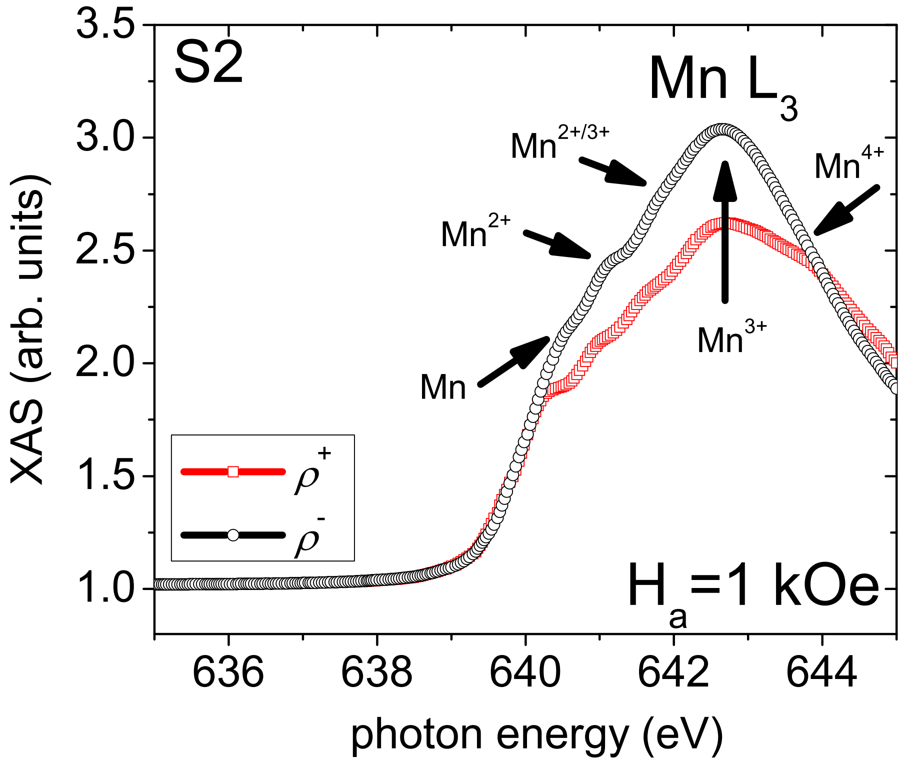

FIG. 2: (Color online) **Mn L edge XAS and XMCD spectrum of specimen S2.** TEY XAS measurements of the Mn $L_{3,2}$ edge with two different photon helicities (ρ^{+} and ρ^{-}) measured at the remanence field after field cooling at 1 kOe for the S2 ML. The multiple arrows indicate the peak positions corresponding to the various oxidation states which are shown within the limited range of energy for clarity.

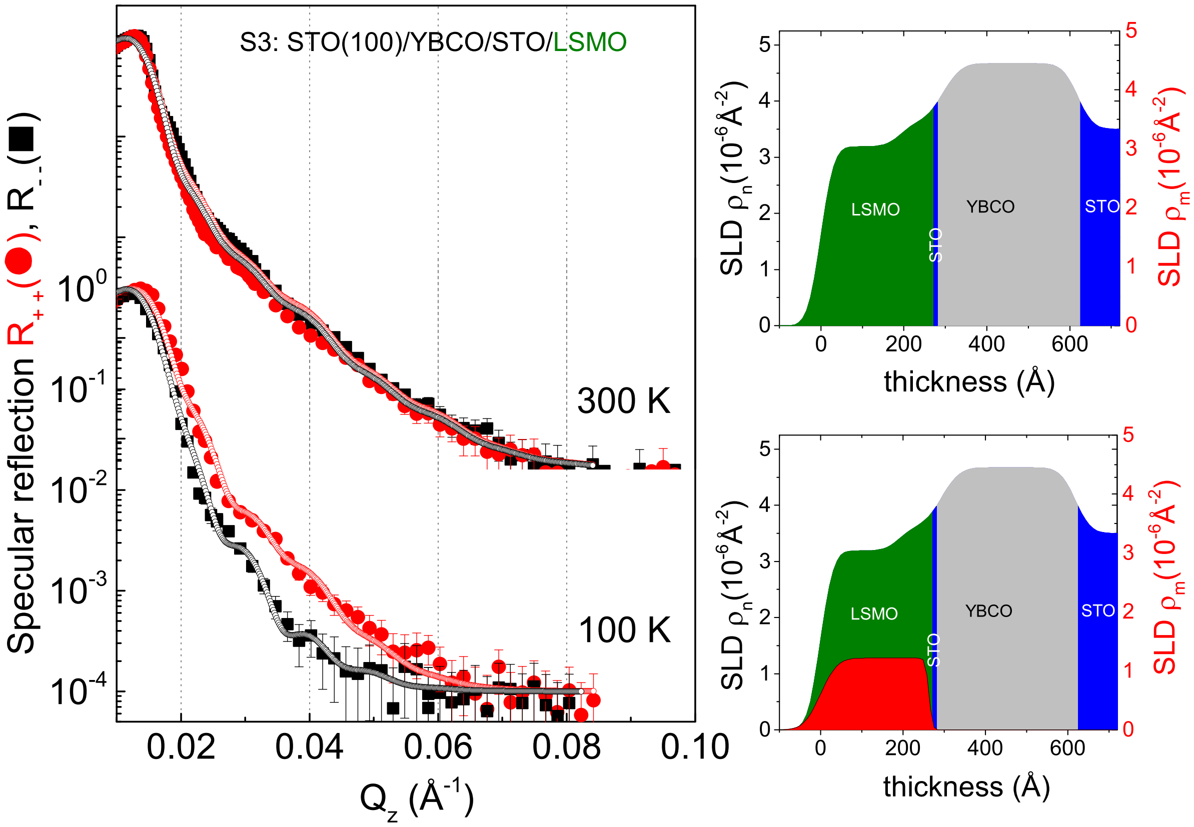

FIG. 3: (Color online) **PNR measurements of specimen S3 at 300 K and 100 K.** Specular neutron reflectivity patterns (solid symbols) along with their best fits (open symbols) as a function of Q_z for the NSF [R_{--} (black) and R_{++} (red)] channels measured at the saturation field $\mathbf{H}_a = +1.0$ kOe for the trilayer sample S3. The measurements are shown for two temperatures, 300 K and 100 K. The nuclear (ρ_n) and magnetic (ρ_m) SLDs versus the thickness of the trilayer are shown alongside.
